# Exploring the Potential of Molecular Hydrogen in Different Heart Failure Models: A Review

**DOI:** 10.3390/ijms262311574

**Published:** 2025-11-28

**Authors:** Daria Kornieieva, Barbora Kalocayova, Jan Slezak, Branislav Kura

**Affiliations:** 1 Centre of Experimental Medicine, Slovak Academy of Sciences, 841 04 Bratislava, Slovakia; daria.kornieieva@savba.sk (D.K.); barbora.kalocayova@savba.sk (B.K.); jan.slezak@savba.sk (J.S.); 2 Institute of Biochemistry and Microbiology, Faculty of Chemical and Food Technology, Slovak University of Technology, 812 37 Bratislava, Slovakia; 3 Department of Pharmacology and Toxicology, Faculty of Pharmacy, Comenius University, 832 32 Bratislava, Slovakia

**Keywords:** cardiac remodeling, cell death, heart failure, inflammation, molecular hydrogen, oxidative stress

## Abstract

Heart failure (HF) is increasing in prevalence in many countries around the world. HF is a complex clinical syndrome characterized by the heart’s inability to pump blood effectively, resulting in significant morbidity and mortality. After an initial cardiac event (e.g., myocardial infarction, valve dysfunction, hypertension, etc.), adaptive mechanisms are activated to preserve cardiac function. Sustained activation of these mechanisms leads to cellular and structural changes involving cardiac remodeling and hypertrophy. This ultimately leads to impaired cardiac contractility and reduced cardiac output, with a 5-year HF-associated mortality rate up to 75%. The current treatment strategies for HF are not sufficient to cover all the underlying complex mechanisms. It has been demonstrated that molecular hydrogen (H_2_) exerts cardioprotective effects via its antioxidant, anti-inflammatory, and anti-apoptotic action. The number of studies exploring beneficial effects of H_2_ in different HF models is increasing. This is the first review summarizing the knowledge in this field. The available literature indicates that H_2_ may be effective in mitigating different HF pathologies via regulating cardiac oxidative stress and inflammation, cardiomyocyte death, and mitochondrial function/cell metabolism, as well as cardiac remodeling, including hypertrophy and fibrosis. As this area of research is still in its infancy, the feasibility and efficiency of H_2_ treatment in different HF types need further investigation.

## 1. Introduction

Heart failure (HF) is increasing in prevalence in many countries worldwide. In Europe, according to the European Society of Cardiology—Heart Failure Association (ESC–HFA), the prevalence of HF was 17.2 cases per 1000 inhabitants (1.7%), with considerable variation between countries; for example, in Lithuania and Germany the prevalence exceeded 30 cases per 1000. Despite improvements in acute myocardial infarction care and more effective therapies for chronic HF, national forecasts project a further 30% rise in HF prevalence by 2035 [1,2].

HF is not a single, specific disease but rather a clinical syndrome in which the heart’s ability to pump or fill with blood is so impaired that characteristic symptoms (such as dyspnea, fatigue, and fluid retention) and objective signs of cardiac dysfunction emerge. HF develops as a result of diverse structural, functional, and molecular abnormalities, and its pathophysiology and etiology vary substantially from patient to patient [3]. The molecular mechanisms underlying HF mainly involve mitochondrial dysfunction (including oxidative stress), lipotoxicity, endoplasmic reticulum stress, defective autophagy, chronic inflammation, programmed cell death, endothelial dysfunction, and impaired contractile signaling. Collectively, these processes drive cardiomyocyte hypertrophy, fibrosis, and depletion, progressively weakening the heart’s pump function [4]. MicroRNAs (miRNAs) have emerged as one of key molecular regulators in the development of HF, linking cellular stress to structural and functional remodeling of the heart. MiRNAs are involved in the pathogenesis of HF at every stage, from endothelial dysfunction and vascular remodeling to the direct regulation of cardiomyocyte metabolism, fibrosis, and apoptosis [5].

Optimal treatment for HF still does not exist due to the complexity of involved pathophysiological mechanisms, which is why the development of new therapeutics remains critically important. This is especially relevant in HF with preserved ejection fraction (HFpEF), which is strongly linked to obesity, insulin resistance, skeletal muscle metabolic inflexibility, and mitochondrial impairment. Current treatments neither restore mitochondrial energetics nor correct metabolic remodeling, leaving a critical driver of disease progression untreated [6]. Molecular hydrogen (H_2_) is considered a therapeutic gas for the treatment of various diseases. Owing to its small size, H_2_ can easily penetrate cell and organelle membranes, which significantly improves its bioavailability. Beyond its selective scavenging of the most harmful radicals (•OH, ONOO^−^), studies have shown that H_2_ exerts antioxidant, anti-inflammatory, anti-apoptotic, mitochondria-protective, metabolic, and immunomodulatory effects [7]. H_2_ delivered in various forms demonstrated cardioprotective effects in different cardiovascular disease models, including reduced ischemia–reperfusion injury, limited infarct size, decreased arrhythmia incidence, and preserved left-ventricular function. Its cardioprotective properties are attributed to various benefits: H_2_ lowers malondialdehyde (MDA) levels and pro-inflammatory cytokines, supports ATP synthesis, and stabilizes the mitochondrial membrane potential [8].

The number of experimental studies dealing with the effects of H_2_ in different HF models is continuously increasing. Therefore, we aimed to prepare a systematic review that summarizes the existing data on the properties of H_2_ in the context of HF, which is currently lacking. The literature search was performed in the PubMed database using keywords “molecular hydrogen” OR “hydrogen” AND “heart failure” OR “isoproterenol” OR “arterial ligation” OR “aortic constriction” OR “monocrotaline” OR “doxorubicin” OR “SHR”. This review may be useful in guiding further H_2_ research in the field of HF treatment.

## 2. Pathophysiology of Heart Failure

HF represents one of the most serious and widespread cardiovascular complications, affecting millions of people worldwide. HF is a syndrome in which the heart’s ability to pump blood or sustain normal pressure is compromised, resulting in symptoms like shortness of breath, fatigue, and swelling of the ankles, as well as clinical signs such as elevated jugular venous pressure, pulmonary crackles, and peripheral edema. Confirming the diagnosis depends on identifying structural or functional cardiac abnormalities. Examples include impaired systolic or diastolic performance following a myocardial infarction, valvular defects, pericardial disease, or disturbances in cardiac rhythm or conduction [9].

HF may develop as a result of various factors, such as increased hemodynamic burden, ischemia, ventricular remodeling, neurohumoral activation, and many others. These mechanisms can lead to deterioration of left ventricular function and other cardiac structures [10]. Patients with HF are typically divided into four categories according to left ventricular contractile performance: HF with reduced ejection fraction (HFrEF), HF with mildly reduced ejection fraction (HFmrEF), HFpEF, and HF with improved ejection fraction (HFimpEF). In HFrEF, the left ventricular ejection fraction (EF) falls below 40%, leading primarily to insufficient stroke volume and cardiac output. HFmrEF is characterized by a persistently stable left ventricular EF in about one-third of patients with HF. This means that over time these patients maintain or even experience an increase in left ventricular EF to a level of approximately 40–45% [11]. By contrast, HFpEF patients maintain a normal EF (over 50%), but exhibit diastolic dysfunction, characterized by impaired ventricular relaxation [12]. HFimpEF refers to HF with improved left ventricular EF in patients who previously had reduced left ventricular EF. Although these patients still take a risk of relapses, they generally have a more favorable prognosis [13].

**Figure 1:**
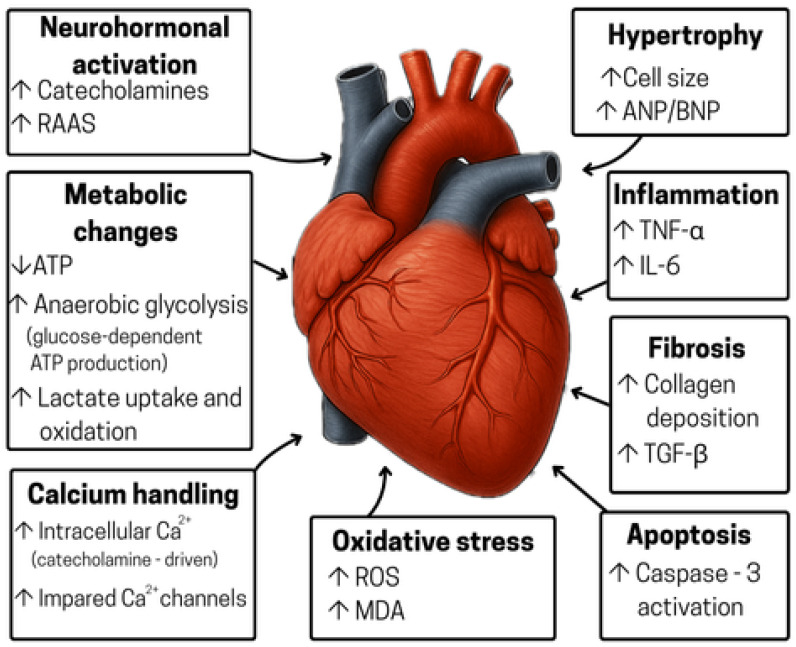
Pathological changes that occur in the heart during HF. RAAS—renin–angiotensin–aldosterone system, ANP—atrial natriuretic peptide, BNP—brain natriuretic peptide, ATP—adenosine triphosphate, TNF-α—tumor necrosis factor alpha, IL-6—interleukin 6, TGF-ß—transforming growth factor beta, ROS—reactive oxygen species, MDA—malondialdehyde.

Immediately after the primary injury, and before clinical symptoms of HF appear, the body initiates several compensatory pathways to sustain cardiac output: catecholamine-driven increases in heart rate and contractility, activation of the renin–angiotensin–aldosterone system (RAAS), release of natriuretic peptides and heightened sympathetic drive [12,14]. Although these compensatory mechanisms temporarily support cardiac function, they also promote cardiomyocyte hypertrophy and fibroblast activation. Next, metabolic reprogramming occurs, the heart shifts from efficient fatty acid oxidation to less efficient, glucose dependent ATP synthesis, exacerbating energy deficits, and oxidative stress [15] (Figure 1).

Before the onset of HF, cardiac hypertrophy develops as an adaptive response to maintain cardiac function. It is characterized by an increase in cardiomyocyte size and thickening of ventricular walls. These changes become maladaptive when the chronic stress persists, leading to ventricular dilation, fall in contractile function and ultimately progressing to HF. Cardiac hypertrophy is accompanied by alterations within cardiomyocytes including calcium handling, metabolism and gene expression (atrial natriuretic peptide—ANP, brain natriuretic peptide—BNP), as well as cell death (e.g., apoptosis), and changes in extracellular matrix (ECM) (fibrosis) and angiogenesis [16].

Dysfunction of cardiac mitochondria is a hallmark of HF and a leading cause of oxidative stress. In addition to producing ATP, mitochondria generate reactive oxygen species (ROS) as byproducts of the respiratory chain. Under normal conditions, ROS support essential cellular functions; however, when their production exceeds elimination (e.g., during HF), oxidative stress arises [17]. An overabundance of ROS and reactive nitrogen species (RNS) damages cells by modifying proteins, lipids, and nucleic acids, ultimately contributing to inflammation, apoptosis, and progression of cardiovascular disease [18]. Several ROS and RNS contribute to HF pathology, including superoxide (O_2_^−^), hydrogen peroxide (H_2_O_2_), hydroxyl radical (·OH), and peroxynitrite (ONOO^−^). Superoxide acts as a primary ROS, generating additional ROS, promoting apoptosis, and driving remodeling by stimulating fibroblast proliferation and metalloproteinase activation. It also reacts with nitric oxide (NO), diminishing NO’s cardioprotective effects and forming peroxynitrite, which damages lipids and proteins, leading to cellular dysfunction and further remodeling. Hydrogen peroxide participates in redox signaling and can give rise to hydroxyl radicals, which are among the most reactive species, causing irreversible damage to lipids, proteins, and DNA and play a central role in the cellular injury seen in HF [19].

Oxidative stress also causes myocardial tissue damage and inflammation, contributing to HF progression. Over time, both the innate and adaptive immune systems become activated, resulting in the release of pro-inflammatory cytokines (e.g., interleukin-6—IL-6, interleukin-1β—IL-1β, tumor necrosis factor-α—TNF-α). These mediators contribute to endothelial dysfunction, oxidative stress, and myocardial injury. Moreover, comorbidities such as diabetes, obesity, or hypertension promote systemic inflammation, thereby contributing to myocardial fibrosis, hypertrophy, and left ventricular dysfunction [20].

Abnormal Ca^2+^ signaling is identified as a form of pathological remodeling in HF. Disrupted Ca^2+^ homeostasis and reduced sarcoplasmic/endoplasmic reticulum Ca^2+^-ATPase (SERCA-2a) activity exacerbate myocardial dysfunction. Consequently, less Ca^2+^ is released during systole, while excess Ca^2+^ remains in the cytosol during diastole, directly contributing to both systolic and diastolic dysfunction of the myocardium [15]. For efficient relaxation to occur, Ca^2+^ needs to be efficiently removed from the cytosol following release. Slowed and incomplete Ca^2+^ removal from the cytosol during HF impairs cardiomyocyte relaxation and promotes hypertrophy and apoptosis signaling [21].

Fibrosis arises in HF when mechanical stress, neurohumoral activation, inflammation, and metabolic disturbances combine to activate cardiac fibroblasts, which remodel the extracellular matrix (ECM), and drive left ventricular hypertrophy. Following myocardial injury, an exaggerated wound-healing response deposits excess collagen types I and III, stiffening the ventricle and impairing diastolic relaxation, while chronic activation of the RAAS and profibrotic mediators such as transforming growth factor beta (TGF-β) and IL-1β promote persistent myofibroblast differentiation and ECM overproduction. Prolonged pressure overload from hypertension or aortic stenosis further increases wall tension, upregulates the expression of pro-fibrotic genes in both cardiomyocytes and fibroblasts, and accelerates collagen crosslinking [22].

Concurrently with pathological fibrosis, programmed cell death pathways are activated in the myocardium during HF. Systemic inflammation promotes increased cardiomyocyte apoptosis by activating caspase 3. Apoptosis is a tightly regulated, caspase-dependent process that preserves membrane integrity while ensuring controlled cell death. It may be activated by intrinsic signals, such as the release of mitochondrial cytochrome c regulated by B-cell lymphoma 2 (Bcl-2) family proteins or by extrinsic signals, specifically via the binding of “death” ligands to TNF/Fas receptors, with subsequent DNA fragmentation and cardiomyocyte destruction. Apoptosis reduces the functional cardiomyocyte pool, triggers compensatory hypertrophy, fibroblast proliferation, and excessive extracellular matrix deposition [15,20].

The heart’s metabolism in HF undergoes extensive changes that reduce mitochondrial oxidative function and cut ATP levels by about 30%, leaving the heart energy starved. Contributing factors include excess ROS and disturbed mitochondrial Ca^2+^ balance, both of which hamper enzyme activity and can initiate cell death. Mitochondrial dynamics are also disrupted, fission is increased, mitophagy is overactive, and fusion is impaired leading to fewer, lower-quality mitochondria. At the same time, transcriptional regulators like peroxisome proliferator-activated receptor gamma coactivator 1-alpha (PGC1α) and peroxisome proliferator-activated receptor alpha (PPARα) are downregulated, weakening mitochondrial biogenesis and fatty-acid oxidation [23]. While healthy hearts rely primarily on fatty acid β-oxidation for ATP production, hypertrophied myocardium switches to glucose utilization to lower oxygen demand and reduce ROS generation [1].

Considering all these interconnected pathophysiological processes, it becomes clear that effective HF therapy requires a comprehensive approach.

### 2.1. microRNAs in Heart Failure

Micro ribonucleic acids (microRNAs, miRNAs) are small, single-stranded, non-coding RNA molecules that typically consist of 21 to 23 nucleotides. These molecules are found in plants, animals, and even some viruses. miRNAs play a crucial role in RNA silencing and the post-transcriptional regulation of gene expression [24]. Additionally, miRNAs are important in predicting and treating HF. Abnormal expression of specific miRNAs is closely associated with HF pathological processes, such as cardiomyocyte apoptosis, myocardial fibrosis, cardiac hypertrophy, and ventricular remodeling. The role of miRNAs is significant in the prognosis of HF [25,26].

Cardiac hypertrophy serves as a vital compensatory mechanism for the heart in response to various pathophysiological stimuli. Several miRNAs are known to be regulated during cardiac hypertrophy [27], with two in particular—miRNA-1 and miRNA-133—playing a crucial role in inhibiting this condition. MiRNA-1 and miRNA-133 are part of the same bicistronic unit and are specifically expressed in skeletal muscle and cardiac myocytes [28]. MiRNA-1 reduces calcium–calmodulin signaling through the calcineurin/nuclear factor of activated T-cells (NFAT) pathway and negatively affects the expression of myocyte-specific enhancer factor 2A (Mef2a) and Gata4, thereby inhibiting cardiomyocyte growth [29]. Similarly, the anti-hypertrophic effect of miRNA-133a was observed through the inhibition of Gq-protein and protein kinase C (PKC) pathways, as well as by offsetting multiple targets involved in calcium signaling, cell growth, and cell development pathways [27,30]. The upregulation of miRNA-30e-5p demonstrates promising anti-hypertrophic effects in cardiomyocytes induced by angiotensin II (Ang II) [31]. Several other miRNAs have been identified as pro-hypertrophic, including miRNA-208, miRNA-21, miRNA-18b, miRNA-195, miRNA-199, miRNA-23, miRNA-24, miRNA-27, and miRNA-9. MiRNA-208 is specific to the heart and is essential for cardiomyocyte hypertrophy, fibrosis, and the expression of beta-myosin heavy chain (β-MHC) in response to stress [32].

There is growing evidence that miRNAs play a significant regulatory role in fibrosis in various organs, including the heart [33]. According to study of Zhao et al. [34], more than 60 miRNAs are directly or indirectly involved in anti- or pro-fibrosis in the cardiac tissues. In particular, several miRNAs, such as members of the miRNA-21 and miRNA-29 families, are increasingly recognized as common regulators of fibrosis across different tissue types [35]. Specifically, miRNA-21 initiates the transformation of inactive cardiac fibroblasts into activated myofibroblasts by targeting the Notch/Jagged1 signaling pathway [36]. Research has shown that miRNA-101a, miRNA-67, and miRNA-15 may function as anti-fibrotic molecules by inhibiting TGFβRI. In a similar manner, miRNA-9, miRNA-590, and miRNA-145 have been noted to play an anti-fibrotic role by targeting TGFβRII [34].

The reduction in functional cardiomyocytes is a primary factor contributing to cardiac remodeling. Three main mechanisms are involved in this process: apoptosis, autophagy, and necrosis. It is widely accepted that miRNAs play a significant role in regulating cardiomyocyte death during the progression of cardiac disease [37,38]. According to current understanding of apoptosis, miRNA-1 facilitates changes in mitochondrial structure by regulating apoptosis-related proteins such as Bcl-2 and Bcl-2-associated X protein (Bax). The overexpression of miRNA-133 reduces the production of ROS and diminishes the oxidative stress response, which helps to decrease cellular apoptosis. Furthermore, miRNA-139 influences the extrinsic death signal of Fas, while miRNA-145 regulates calcium overload in the endoplasmic reticulum. Both of these actions have a regulatory effect on cardiomyocyte apoptosis [39]. The signaling pathways linking autophagy and apoptosis are intricate and often occur simultaneously. By inhibiting both autophagy and apoptosis through the protein kinase B (Akt)/mammalian target of rapamycin (mTOR) pathway, miRNA-223 protects heart tissue from ischemic damage and hypoxia in myocardial cells [40]. Several miRNAs are implicated in heart necrosis. These miRNAs can either promote or inhibit necrosis, and their involvement is often linked to targeting specific proteins involved in the necrotic cell death pathways. Long non-coding RNA H19 has been shown to directly bind to miRNA-103/107, thereby regulating the expression of Fas-associated death domain protein (FADD) and influencing necrosis [41]. MiRNA-155 offers a mechanism to block necrosis by targeting receptor interacting protein 1 (RIP1) independently on the activation of the Akt pro-survival pathway [42].

### 2.2. Rodent Models of Heart Failure

**Figure 2:**
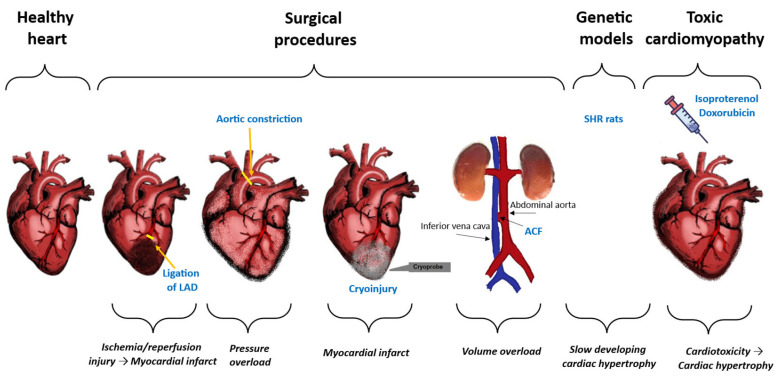
In studies involving rats, heart failure (HF) can be induced through various methods, including surgical methods as arterial ligation (LAD; Left Anterior Descending artery), aortic constriction, or cryogenic damage with a metal probe cooled in liquid nitrogen. Aortocaval fistula (ACF) in rats serves as a unique model for studying volume-overload congestive HF. Genetic models of HF include spontaneous hypertensive rats (SHR) which mimic slow-developing cardiac hypertrophy. Other models of HF that utilize drug administration to induce HF in experimental rodent models include direct toxicity to myocardial tissue caused by agents such as doxorubicin or isoproterenol.

Over the past few decades, numerous animal models have been developed to replicate various mechanisms that contribute to HF. Despite their limitations, these models have significantly advanced understanding of the different causes of HF and have helped clarify the underlying mechanisms. These models employ surgical techniques, pharmacological methods, and genetic engineering to study HF [43,44,45] (Figure 2).

Animal models of HF can be developed using various surgical procedures, including arterial ligation, aortic constriction, or cryogenic damage with a metal probe cooled in liquid nitrogen [46]. Aortocaval fistula in rats serves as a unique model for studying volume-overload congestive HF and cardiac hypertrophy [47,48]. Currently, the ligation of the left anterior descending artery has become the preferred model for inducing ischemia because the pathology seen in this model relates to clinically relevant observations [49,50]. Permanent coronary artery loss results in a complete blockage of blood flow and irreversible hypoxia, which puts most of the surrounding area at risk of infarction and leads to the formation of a large, permanent scar in the myocardium. This damaged region is prone to pathological remodeling, which contributes to the progression of HF [51]. A model of aortic constriction is effective for inducing pressure overload and examining left ventricular hypertrophy. This method enhances the pressure within the left ventricle by partially constricting the aorta [52]. Cryoinjury is a technique used to create a myocardial infarction rat model. It involves applying a cold probe to the surface of the heart, usually targeting the left ventricle, to freeze and damage a small area of heart tissue. This injury triggers a local inflammatory response and leads to scar formation, which mimics the physiological changes observed in human myocardial infarction [53].

In contrast to surgical techniques, HF models have been developed that use drug administration to induce HF in experimental rodent models. This approach involves the direct toxicity to myocardial tissue caused by substances such as isoproterenol, doxorubicin, angiotensin II, monocrotaline, ethanol, or homocysteine [54]. The isoproterenol-induced HF model is a commonly used experimental method to investigate HF in animal subjects, particularly in mice and rats. Isoproterenol-induced cardiac hypertrophy serves as a reliable, reproducible, and well-characterized model for studying cardiac hypertrophy, which is linked to arrhythmia, myocyte loss, and fibrosis, ultimately leading to HF. As a non-selective beta-adrenergic agonist, isoproterenol induces cardiac injury and dysfunction by increasing heart rate and contractility, thereby mimicking the effects of stress on the heart [55,56]. Cardiotoxicity is the most significant and dose-limiting side effect of doxorubicin (also known as Adriamycin), an anticancer drug. Doxorubicin-induced cardiac toxicity is characterized by thinning of the ventricular walls and dilation of the left ventricular chamber. Various pathogenic mechanisms have been identified as contributing to doxorubicin-induced dilated cardiomyopathy, including mitochondrial dysfunction, apoptosis of cardiac myocytes, and alterations in calcium handling [57,58]. Non-surgical models of left ventricular failure include Ang II infusion that mimic slow-developing cardiac hypertrophy as seen in the progression of HFpEF [46]. Monocrotaline model is commonly used to investigate the cellular mechanisms related to the development of right HF. Monocrotaline damages the endothelial cells in the lungs, leading to pulmonary hypertension, which eventually results in right ventricular hypertrophy and failure [59,60]. In rats, the consumption of ethanol can result in different types of HF, including alcoholic cardiomyopathy. This condition is marked by reduced cardiac function and structural changes in the heart. Chronic exposure to ethanol disrupts myocardial protein synthesis, which further impairs heart function. Additionally, ethanol can lead to the apoptosis of cardiomyocytes and dilation of the left ventricle [61,62]. Supplementing rats’ diet with homocysteine can also lead to ventricular dysfunction. This was evidenced by a significant increase in collagen content and echocardiographic changes, including an increase in the thickness of the posterior wall and the interventricular septum [63]. Streptozotocin (STZ)-induced HF, commonly referred to as diabetic cardiomyopathy, is a significant complication associated with type 1 diabetes mellitus in rodent models. This condition can present as both HFrEF and HFpEF. In these models, STZ induces hyperglycemia and disrupts myocardial function, resulting in HF symptoms such as bradycardia and decreased blood pressure [64,65,66].

Rodent genetic models of HF include spontaneously hypertensive rats (SHR), which develop HF over time. Spontaneous hypertension is a natural model of pressure overload, leading to HF with aging [67]. Spontaneously hypertensive HF-prone rats (SHHF) develop HF earlier than the SHR strain, with loss of cardiac function starting at the age of 15 months [68]. Dahl-salt-sensitive rats are characterized by hypersensitivity to sodium intake. When administered with high-salt diet, they develop left ventricular hypertrophy and later decompensate HF with marked ventricular dilation [69]. Rodent models of type 2 diabetes include the Zucker fatty rat, as well as db/db and ob/ob mice, which have a mutation in the leptin receptor. These models are engineered to mimic HFpEF and are used to study the link between diabetes and HF [70].

## 3. Potential of Molecular Hydrogen Therapy in Heart Failure—Mechanistic Insight

H_2_ is the smallest and lightest ubiquitous molecule. Owing to its small size, H_2_ can penetrate through any biological barrier, including the blood–brain barrier, and diffuse throughout the body, as has been demonstrated in studies with rats and pigs [71,72]. In cells, H_2_ can be rapidly distributed into the cytosol and organelles, and it can enter the mitochondria and nucleus, what makes it available directly at the site of action [73]. Another advantage is the broad range of H_2_ administration methods, e.g., drinking of H_2_-rich water, inhalation of H_2_-rich air, or injection of H_2_-rich saline [74]. H_2_ gas is explosive in a reaction with O_2_ in the concentration range 4–75% (*v*/*v*); therefore, medical applications of H_2_ need to take place outside this range. Nevertheless, even the concentrations of H_2_ up to 4% exhibit beneficial effects [75]. The trials conducted so far confirm the safety and the nearly absent toxicity of H_2_ administration in humans [76].

**Figure 3:**
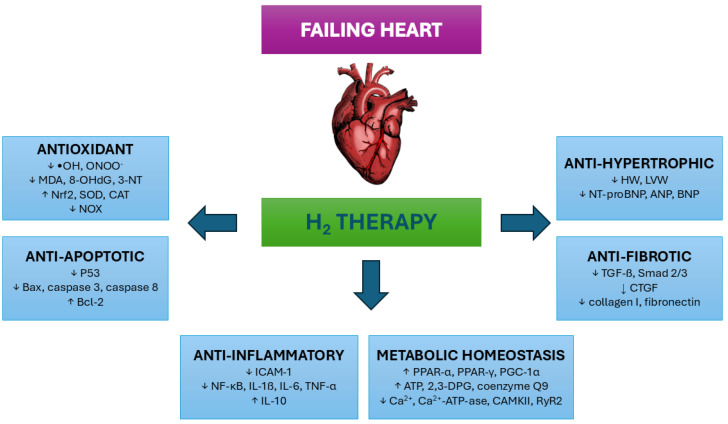
Possible mechanisms of H_2_ cardioprotective action during heart failure onset and progression. •OH—hydroxyl radical, ONOO^−^—peroxynitrite, MDA—malondialdehyde, 8-OHdG—8-hydroxy-2-deoxyguanozine, 3-NT—3-nitrotyrosine, Nrf2—nuclear factor erythroid 2-related factor 2, SOD—superoxide dismutase, CAT—catalase, NOX—nicotinamide adenine dinucleotide phosphate oxidase, Bax—B-cell lymphoma 2-associated X protein, Bcl-2—B-cell lymphoma 2, ICAM-1—intercellular adhesion molecule 1, NF-κB—nuclear factor kappa B, IL-1β—interleukin 1 beta, IL-6—interleukin 6, TNF-α—tumor necrosis factor alpha, IL-10—interleukin 10, PPAR-α—peroxisome proliferator-activated receptor alpha, PPAR-γ—peroxisome proliferator-activated receptor gamma, PGC-1α—peroxisome proliferator-activated receptor-gamma coactivator-1 alpha, ATP—adenosine triphosphate, 2,3-DPG—2,3-diphosphoglyceric acid, Ca^2+^-ATP-ase—calcium adenosine triphosphatase, CAMKII—calcium/calmodulin-dependent protein kinase II, RyR2—ryanodine receptor 2, TGF-β—transforming growth factor beta, CTGF—connective tissue growth factor, HW—heart weight, LVW—left ventricle weight, NT-proBNP—N-terminal B-type natriuretic peptide precursor, ANP—atrial natriuretic peptide, BNP—brain natriuretic peptide.

Biological properties of H_2_, that may be involved in the protection of heart during HF onset and progression, include its selective antioxidant properties and anti-inflammatory action, it can also reduce cell apoptosis and improve cell metabolism as well as exerts anti-hypertrophic and anti-fibrotic effect [8] (Figure 3).

Conventional antioxidants, such as vitamin E, failed to demonstrate therapeutic benefits for HF in preclinical models. This may be attributed to their inability to selectively target mitochondria, where oxidative stress is most pronounced during HF, or their broad scavenging of ROS that may disrupt essential cellular processes [77]. In this sense, H_2_ may be a suitable antioxidant molecule for HF as it selectively reacts only with the most harmful radicals, like hydroxyl (•OH) and peroxynitrite (ONOO−), without affecting ROS with a physiological role [73]. Due to the unique small size of H_2_ molecule, the neutralization of ROS can occur in any cell compartment, including mitochondria [78]. It has been demonstrated that the administration of H_2_ effectively decreased oxidative stress markers, like MDA, 8-hydroxy-2′-deoxyguanosine (8-OHdG) or 3-nitrotyrosine, in different HF models [79,80,81]. H_2_ can also exert the antioxidant effect via the induction of the nuclear factor erythroid 2-related factor 2 (Nrf2) pathway, thereby activating endogenous antioxidant enzymes [82]. In rats, supplementation with H_2_ enhanced superoxide dismutase (SOD) and catalase (CAT) activity under HF conditions [80,83,84,85]. Moreover, H_2_ has been shown to inhibit enzymes producing ROS, such as nicotinamide adenine dinucleotide phosphate oxidase (NOX) [80,84].

Anti-inflammatory action of H_2_ has been proved in different experimental disease models, including HF [86,87]. At the early stage of inflammatory reaction, H_2_ can decrease the infiltration of neutrophils and macrophages by downregulating the expression of adhesion molecules (e.g., intercellular adhesion molecule 1—ICAM-1) and chemokines [88]. Besides that, H_2_ can affect the inflammatory process by regulating nuclear transcription factors and downstream pro-inflammatory cytokines. It has been revealed that H_2_ can modulate inflammatory signaling pathways, including the nuclear factor kappa B (NF-κB) pathway [89]. H_2_ decreased inflammatory markers IL-6 and TNF-α in the serum and/or cardiac tissue of rats with isoproterenol-induced myocardial infarction [85]. H_2_ exerted its cardioprotective effect on rat’s heart damaged by doxorubicin via lowering the inflammatory molecules like TNF-α, IL-6, and IL-1ß [87]. In addition, H_2_ increases the expression of anti-inflammatory molecules like IL-10 [90].

A key hallmark of HF is the loss of cardiomyocytes via apoptosis, what leads to the loss of myocardial tissue and contributes to disease development [91]. It has been shown that H_2_ effectively decreases apoptosis and other forms of programmed cell death. In rats with isoproterenol-induced myocardial infarction, H_2_ treatment improved cardiac function, reduced infarct size and apoptosis. P53 has been identified as a transcription factor associated with anti-apoptotic effects of H_2_ [79]. The application of H_2_-rich saline via intraperitoneal injection in rats treated with doxorubicin exerted cardioprotective effects via lowering pro-apoptotic molecules (Bax, caspase 8, caspase 3) as well as increasing anti-apoptotic molecules (Bcl-2) [87]. Similar apoptosis-reducing effects of H_2_ were seen in a rat model of isoproterenol-induced HF. Intraperitoneal injection of H_2_-rich saline ameliorated myocardial injury via inhibition of cardiomyocyte apoptosis by upregulating Bcl-2, and downregulating Bax [92]. Inhibition of apoptosis by H_2_ may involve activation of various signaling pathways, e.g., phosphoinositide 3-kinase/protein kinase B (PI3K/AKT) pathway [93].

Cardiac hypertrophy typically emerges before the onset of HF as an adaptive response to maintain cardiac function after preceding increased workload or cardiac insult [16]. Administration of H_2_-rich saline mitigated cardiac hypertrophy and HF induced by the pressure overload in rats as shown by lower heart and atrial weights [94]. Hypertrophy was also reduced in the heart of rats with isoproterenol-induced HF after H_2_ treatment. The authors detected decreased heart and left ventricle weights as well as lowered levels of a biomarker N-terminal B-type natriuretic peptide precursor (NT-proBNP) in the plasma [92]. Intraperitoneal injection of H_2_-rich saline prevented isoproterenol-induced cardiac hypertrophy and improved cardiac function in mice. This was reflected by lowered heart weight/body weight ratio as well as reduced ANP and BNP levels in the left ventricles [81].

The anti-fibrotic effect of H_2_ has been demonstrated in various HF models. H_2_ administered by intraperitoneal injection prevented interstitial fibrosis and the progression of HF induced by pressure overload (aortic constriction) in rats. H_2_ treatment decreased the phosphorylation of p38 mitogen-activated protein kinase (MAPK) and Smad2/3, and the expression of TGF-β1 and connective tissue growth factor (CTGF), which were accompanied by reduced hydroxyproline content, collagen I (Col I) and fibronectin 1 (FN1) mRNA levels [84]. After inhalation of H_2_-rich air, decrease in atrial fibrosis was measured in rats with atrial fibrillation induced by Ang II. H_2_ reduced Ang II-mediated atrial fibrosis through inhibiting TGF-β1/Smad2/3 pathway [80]. Similar effects were seen in another study, where the authors found that H_2_-rich saline mitigated atrial fibrosis induced by pressure overload in rats. This was possibly achieved via inhibition of the Janus kinase/signal transducer and activator of transcription protein (JAK-STAT) signaling pathway [94]. In a monocrotaline-induced pulmonary hypertension model (representing right ventricle HF), inhalation of H_2_-rich air reduced fibrosis of lung tissue via lowering TGF-ß expression levels [95]. Intraperitoneal injection of H_2_-rich saline reduced collagen deposition and myocardial fibrosis in rats with isoproterenol-induced HF [92].

Mitochondria ensure the homeostasis of energy metabolism. When disrupted during HF, metabolic remodeling occurs, leading to a shift in metabolic substrate utilization from free fatty acid to glucose oxidation, insufficient ATP production and Ca^2+^ overload [96]. The in vivo and in vitro assays showed that H_2_ promoted fatty acid β-oxidation by modulating the activation of adenosine monophosphate-activated protein kinase (AMPK) signaling and PPARα/γ [97]. Other authors found that H_2_ stimulates the expression of PGC-1α to enhance fatty acid metabolism. H_2_ has been proven to stimulate energy production. H_2_ inhalation increased ATP and 2,3-diphosphoglyceric acid (2,3-DPG) levels in erythrocytes of rats with induced chronic HF thereby enhancing oxygen delivery to tissues [98]. In another study with rats undergoing heart transplantation, H_2_ treatment was also associated with increased ATP levels in grafts and increased activity of respiratory chain enzymes in mitochondria [99]. Gvozdjáková et al. [100] demonstrated that consumption of H_2_-rich water resulted in stimulated mitochondrial respiratory chain function, increased levels of ATP and coenzyme Q9 in the rat myocardium. H_2_ has also been shown to regulate calcium levels. In a different study, H_2_-rich water administration prevented elevation of intracellular Ca^2+^ levels in a model of ischemic brain injury [101]. H_2_ contributes to calcium homeostasis also via its effects on different enzymes and receptors involved in calcium signaling, e.g., Ca^2+^—ATPase activity [85], or phosphorylation of CaMKII enzyme and ryanodine receptor 2 (RyR2) [80].

## 4. Experimental Evidence for Molecular Hydrogen Effects in Different Heart Failure Models

As stated above, HF is a complex syndrome that involves many pathophysiological mechanisms. There is no single HF model that could cover such a complexity of underlying mechanisms. Experimental studies exploring the effects of H_2_ on different HF models are continuously increasing. Their results are summarized below as well as in Table 1.

### 4.1. In Vitro Studies

The prevailing *in vitro* model to study the effect of H_2_ was a model of cardiac cells damaged by isoproterenol. Chen et al. [92] conducted in vitro experiments using H9c2 cardiomyocytes incubated with isoproterenol. Cultivation of isoproterenol-treated H9c2 cells in H_2_-rich medium improved the survival and proliferation of cells, reduced the cell surface area, indicating a protective effect against hypertrophy. Furthermore, H_2_-rich medium alleviated apoptosis via lowering Bax levels and enhancing Bcl-2 expression in isoproterenol-treated cells. In another study, the authors revealed that isoproterenol-induced excessive autophagy in H9c2 cardiomyocytes was blocked by pretreatment with H_2_-rich medium [102]. Anti-hypertrophic effects of H_2_ were confirmed in H9c2 cardiomyocytes treated with isoproterenol. H_2_ lowered the excessive expression of NOX and the accumulation of ROS, attenuated the decrease in matrix metalloproteinase (MMP), and inhibited ROS-sensitive extracellular signal-regulated kinase 1/2 (ERK1/2), p38, and c-Jun N-terminal kinase (JNK) signaling pathways [81].

Other in vitro models were also employed to study H_2_ effects in HF-related conditions. In vitro experiments were performed using cardiotrophin-1 (CT-1)-induced hypertrophy in cultured neonatal rat cardiomyocytes. The addition of H_2_-rich saline to the medium mitigated cells’ hypertrophy, as demonstrated by lowered cell surface area. In addition, H_2_ significantly downregulated the expression of IL-6 and JAK/STAT3 signaling pathway expression in hypertrophic cells [94]. To demonstrate H_2_ potential for preventing and treating atrial fibrillation, HL-1 atrial cardiomyocytes and rat fibroblasts were treated with Ang II. Decrease in ROS and NLRP3 inflammasome was detected in Ang II-treated HL-1 cells that were cultivated under 75% H_2_. At the same time, improvement in calcium handling and K^+^ channel function was also detected in this model when incubated under 75% H_2_. H_2_ treatment suppressed proliferation and migration of atrial fibroblasts cultivated in the presence of Ang II. H_2_ inhibited TGF-β1 secretion and activation and decreased Ang II-induced atrial fibrosis through suppressing TGF-β1/Smad2/3 pathway [80]. In another study, H_2_ treatment attenuated doxorubicin-induced injury in H9c2 cardiomyocytes. Incubation of cardiomyocytes in H_2_-saturated medium inhibited doxorubicin-induced apoptosis, as measured by decreased expression of Bax, cleaved caspase-3 and -9 as well as upregulation of Bcl-2. It has been demonstrated that H_2_ treatment attenuated doxorubicin-induced cardiomyocyte injury by activating autophagy via the AMPK/mTOR pathway [103].

### 4.2. In Vivo Studies

The most frequently used *in vivo* model to study the effect of H_2_ was a model of heart damage induced by isoproterenol. Chi et al. [79] aimed to investigate the possible mechanism of H_2_ inhalation in delaying the progression of chronic HF. The chronic HF model was established by subcutaneous injection of isoproterenol in rats. H_2_ inhalation (2% in air) improved heart function with significant attenuation of oxidative stress damage and apoptosis. P53 has been identified as a transcription factor potentially associated with antioxidant and anti-apoptotic effects of H_2_. In another study, it has also been demonstrated that administration of H_2_-rich saline shows cardioprotective effects in a rat model of isoproterenol-induced HF. This was evidenced by reduced myocardial fibrosis and cardiac hypertrophy after H_2_ administration. Markers of tissue damage were also lowered [creatine kinase—myocardial band (CK-MB), troponin-I and NT-proBNP]. Furthermore, cardiomyocyte apoptosis was inhibited [92]. H_2_ was able to inhibit autophagy mediated by isoproterenol in mice in vivo, as was documented by suppressed expression levels of Beclin1, autophagy-related gene 7 (Atg7) and microtubule-associated protein 1 light chain 3B (LC3B II). H_2_ as well mitigated hypertrophy induced by isoproterenol in mice hearts, as indicated by decreasing HW and HW/BW ratio [102]. Zhang et al. [81] investigated the effects of H_2_ on cardiac hypertrophy induced by isoproterenol in mice. H_2_ intraperitoneal injection decreased hypertrophic markers ANP and BNP and HW/BW ratio as well as alleviated the impaired left ventricular function. H_2_ exerted its protective effects partially through blocking ROS-sensitive ERK1/2, p38, and JNK signaling pathways. In another study, isoproterenol was used to induce myocardial infarction in rats. To mitigate the heart damage, rats were given different doses of H_2_-rich saline or vitamin C. H_2_-rich saline decreased oxidative stress parameters MDA and 8-OHdG, enhanced SOD and Na^+^/K^+^-ATPase activity, lowered Ca-ATPase activity and decreased inflammatory markers IL-6 and TNF-α in the serum and/or cardiac tissue of rats. H_2_-rich saline pretreatment also reduced infarct size and improved left ventricle function [85].

H_2_ administered by intraperitoneal injection as saturated saline prevented interstitial fibrosis and the progression of HF induced by pressure overload (aortic constriction) in rats. This was evidenced through its antioxidant properties and via suppression of TGF-β1 signaling. H_2_ treatment decreased the level of oxidative stress parameters ROS, MDA, and NOX, while the activity of the antioxidant enzyme SOD was increased. Administration of H_2_ was associated with decreased expression of TGF-β1 and CTGF. These molecular changes coincided with lower hydroxyproline content and downregulation of Col I and FN1 mRNA levels [84]. Pressure overload was induced in rats by abdominal aortic constriction. Administration of H_2_-rich saline mitigated cardiac hypertrophy and HF induced by the pressure overload and reduced atrial fibrosis and atrial fibrillation possibly via JAK-STAT signaling pathway. The authors observed better results with higher doses of H_2_-rich saline (6 mL/kg) [94].

Atrial fibrillation is one of the main factors leading to HF. Experimental rats were given Ang II at low doses (1080 μg/kg/24 h) via osmotic minipumps continuously over 28 days. Rats that inhaled 2% H_2_ in air (6 h per day) for 28 days showed decreased susceptibility to atrial fibrillation. At the same time, H_2_ decreased atrial fibrosis (TGF-ß1), oxidative stress (MDA) and inflammation and improved Ca^2+^ handling via inhibition of phosphorylation of Ca channels [80]. Ventricular fibrillation was induced by transcutaneous electrical epicardial stimulation in rats with subsequent cardiac arrest. The results of the study revealed that inhalation of H_2_ gas (2% H_2_ in O_2_) during cardio-pulmonary resuscitation and for 2 h after the return of spontaneous circulation improved the survival rate of rats experiencing cardiac arrest after ventricular fibrillation. H_2_ prevented a rise in left ventricular end-diastolic pressure and increase in serum IL-6. At the same time, H_2_ inhalation ameliorated oxidative myocardial injury, as both 8-OHdG- and 4-hydroxynonenal (4-HNE)-positive cardiomyocytes were markedly suppressed [104].

He et al. [105] explored protective effects of H_2_ on monocrotaline-induced pulmonary hypertension ultimately leading to right-sided HF. H_2_ was administered to rats either orally as H_2_-rich water or via intraperitoneal injection of H_2_-rich saline. Both forms of administration effectively decreased pulmonary hypertension and inflammatory response as well as mitigated right ventricular hypertrophy. The protective effect of H_2_ was attributed to its antioxidant and anti-inflammatory action. In a similar model Kuropatkina et al. [95] found that inhalation of 4% H_2_ in air for 21 days reduces inflammation (mast cells) and fibrosis (TGF-ß) of lung tissue during monocrotaline-induced pulmonary hypertension.

Doxorubicin is one of the effective anticancer treatments, but its use is limited due to cardiotoxicity that might lead to HF. Gao et al. [87] found that intraperitoneal injection of H_2_-rich saline ameliorated the mortality, cardiac dysfunction and histopathological changes caused by doxorubicin in rats. H_2_ exerted its protective effect via lowering oxidant (MDA, ROS), inflammatory (TNF-α, IL-6, IL-1ß) and apoptotic molecules (Bcl-2, Bax, caspase 8, caspase 3). In another study, chronic heart injury model was also established by intraperitoneal injection of doxorubicin in rats. H_2_ inhalation improved doxorubicin-induced decline in cardiac function (left ventricular diameter, left ventricular ejection fraction, fractional shortening) as well as pathological structural abnormalities in rats. Cardiac injury markers lactate dehydrogenase (LDH), CK-MB, cTnI, BNP as well as apoptotic markers were decreased after H_2_ treatment. H_2_ inhalation activated autophagy through AMPK/mTOR pathway, thereby protected against myocardial injury induced by doxorubicin [103].

In another study, the effects of H_2_ inhalation on the functional states of red blood cells (RBCs) in rats with chronic HF were explored. Chronic HF was induced by intraperitoneal injection of 1% adrenaline. Application of H_2_ via inhalation (2% in air) improved microcirculation and oxygen transport function of blood. This was concluded based on the increased electrophoretic mobility of erythrocytes (EPM) and decreased level of their aggregation as seen after 14 days of H_2_ treatment. At the same time, H_2_ increased ATP and 2,3-diphosphoglyceric acid (2,3-DPG) levels in erythrocytes thereby enhancing oxygen delivery to tissues. Oxidative stress and hematological parameters were also improved in a group receiving H_2_ [83].

**Table 1:** Experimental studies exploring the effects of molecular hydrogen in different heart failure models. HF—heart failure, H_2_—molecular hydrogen, ISO—isoproterenol, H9c2 cells—cardiomyocyte cell line, LVESD—left ventricular end-systolic dimension, LVEDD—left ventricular end-diastolic dimension, RAVD—right atrium vertical diameter, RATD—right atrium transverse diameter, IVS—interventricular septum, RV—right ventricle, EF—ejection fraction, FS—fractional shortening, E/A—mitral E/A peak velocity, BNP—brain natriuretic peptide, 8-OHdG—8-hydroxy-2′-deoxyguanosine, MDA—malondialdehyde, Bcl-2—B-cell lymphoma 2, Bax—Bcl-2 associated X-protein, p53—transcription factor p53, IP—intraperitoneal, EPM—electrophoretic mobility, ATP—adenosine triphosphate, 2,3-DPG—2,3-diphosphoglyceric acid, CAT—catalase, RBC—red blood cell count, Hg—hemoglobin, MCV—mean corpuscular volume, Col I—collagen I, FN1—fibronectin 1, TGF-β1—transforming growth factor β1, CTGF—connective tissue growth factor, p-Smad2/3—phosphorylated Smad2/3, NOX—nicotinamide adenine dinucleotide phosphate oxidase, p-p38 MAPK—phosphorylated p38 mitogen-activated protein kinase, CPR—cardio-pulmonary resuscitation, ROSC—return of spontaneous circulation, IL—interleukin, LVEDP—left ventricular end-diastolic pressure, 4-HNE—4-hydroxynonenal, HW—heart weight, BW—body weight, AW—atrial weight, LVW—left ventricle weight, HR—heart rate, LVAWd—LV anterior wall thickness at end-diastole, LVPWd—LV posterior wall thickness at end-diastole, LVD—left ventricular diameter, ROS—reactive oxygen species, TNF-α—tumor necrosis factor α, LC3—microtubule-associated protein 1 light chain 3, AMPK—adenosine monophosphate-activated protein kinase, mTOR—mammalian target of rapamycin, AngII—angiotensin 2, AF—atrial fibrillation, NLRP3—Nucleotide-Binding Domain, Leucine-Rich–Containing Family, Pyrin Domain–Containing-3, ASC—Apoptosis-associated speck-like protein containing a caspase recruitment domain (CARD), CaMKII—Ca^2+^/calmodulin-dependent protein kinase II, RyR2—Ryanodine receptor 2, α-SMA—alpha-smooth muscle actin, Smad—Mothers against decapentaplegic homolog, MCT—monocrotaline, mPAP—mean pulmonary arterial pressure, RVHI—right ventricular hypertrophy index, ANF—atrial natriuretic factor, LVESV—left ventricular end-systolic volume, LVEDV—left ventricular end-diastolic volume, TB—tibia length, CK-MB—creatine kinase-MB, cTn-I—cardiac troponin-I, NT-proBNP—N-terminal B-type natriuretic peptide precursor, p-cTn-I—phosphorylated cardiac troponin-I, Atg7 -autophagy-related protein 7, LC3B—microtubule-associated protein 1 light chain 3β, MMP—matrix metalloproteinase, ERK—extracellular signal-regulated kinase, JNK—c-Jun N-terminal kinase, AST—aspartate aminotransferase, LVSP—left ventricular systolic pressure, +dP/dt max—maximal rate of pressure rise, -dP/dt max—maximal rate of pressure fall.

HF Model	H_2_ Administration	H_2_ Effects	Reference
**ISO** (85, 170, 340 mg/kg bw) subcutaneous injection (rats) Menadione (10 μM) (H9c2 cells)	2% H_2_ in air inhalation 12 h daily for 14 days H_2_ saturated medium (min. 0.6 mM)	↓ LVESD, LVEDD, RAVD, RATD, IVS, RV ↑ EF, FS, E/A ↓ BNP ↓ fibrosis ↓ 8-OHdG, MDA ↓ apoptosis, Bax, cleaved caspase-3 ↓ p53	Chi et al. [79]
**ISO** (10 mg/kg/day) for 14 days subcutaneous injection (rats) ISO (25 μg/mL) for 24 h (H9c2 cells)	H_2_ saturated saline (0.6 mmol/L) IP injection (10 mL/kg/d) for 14 days H_2_-rich medium (15, 50, or 150 μM)	↓ LVEDD, LVESD, LVESV, LVEDV ↑ EF, FS ↓ hypertrophy, HW/BW, LVW/BW, LVW/TL ↓ fibrosis ↓ CK-MB, cTn-I, NT-proBNP, p-cTn-I ↓ apoptosis, Bax ↓ Bcl-2	Chen et al. [92]
**ISO** (0.5 mg/100 g/day) for 7 days subcutaneous injection (mice) ISO (10 μM) (H9c2 cells)	HRS (1 mL/100 g/day) IP injection 7 days pretreatment 7 days treatment H_2_-rich medium	↓ HW/BW ↑ Beclin1, Atg7, LC3B II	Zhang et al. [102]
**ISO** (0.5 mg/100 g/day) for 7 days subcutaneous injection (mice) ISO (10 μM) (H9c2 cells)	HRS (1 mL/100 g/day) IP injection 7 days pretreatment 7 days treatment H_2_-rich medium	↓ ANP, BNP, HW/BW ↓ LVESD, LVEDD ↑ FS ↓ ROS, 3-nitrotyrosine, p67, MMP ↓ phosphorylation of ERK1/2, p38, JNK	Zhang et al. [81]
**ISO** (200 mg/kg bw) subcutaneous injection (rats)	HRS (5, 7.5, 10 mL/kg bw) IP injection	↓ infarct size ↓ CK-MB, AST activity ↑ LVSP, +dP/dt max, -dP/dt max ↓ LVEDP ↓ MDA, 8-OHdG ↑ SOD activity ↓ IL-6, TNF-α ↑ Na^+^-K^+^-ATPase ↓ Ca2+-ATPase	Jing et al. [85]
abdominal **aortic constriction** (rats)	H_2_ saturated saline IP injection 10 mL/kg daily for 16 weeks	↓ LVESD, LVEDD ↑ EF, FS ↓ hydroxyproline, Col I, FN1, TGF-β1, CTGF, p-Smad2/3 ↓ ROS, MDA, NOX2, NOX4 ↑ SOD activity ↓ p-p38 MAPK	Yang et al. [84]
abdominal **aortic constriction** (rats) Cardiotrophin-1 (0.1 nM) (neonatal rat cardiomyocytes)	H_2_ saturated saline IP injection 3 or 6 mL/kg HRS daily for 6 weeks H_2_ saturated medium	↓ HW/BW, AW/BW, LVW/BW ↑ AW/HW ↓ HR, LVAWd, LVPWd ↓ atrial fibrillation ↓ atrial fibrosis ↓ IL-6, JAK, STAT3	Wang and Pan [94]
**Ang II** (1080 μg/kg/24 h) Daily for 28 days Osmotic minipumps (rats) Ang II (1.0μM) HL-1 atrial cardiomyocytes rat fibroblasts	2% H_2_ in air inhalation 6 h a day for 28 days 75% H_2_	↓ AF susceptibility and duration ↓ left atrial diameter and area ↓ MDA, NOX4 ↑ SOD activity ↓ NLRP3, ASC, IL-1ß ↓ caspase 1 ↓ p-CaMKII, p-RyR2 ↓ TGF-ß1, α-SMA, Col I, III, p-Smad2, p-Smad3	Zhang et al. [80]
transcutaneous **electrical epicardial stimulation** (rats)	2% H_2_ in O_2_ inhalation during CPR and 2 h after ROSC	↑ survival ↓ IL-6 ↓ LVEDP ↓ fibrosis ↓ 8-OHdG- and 4-HNE-positive cells	Hayashida et al. [104]
**MCT** (80 mg/kg) IP injection (rats)	H_2_ saturated water (0.6 mmol/L) H_2_ saturated saline (0.6 mmol/L) IP injection for 28 days	↓ mPAP ↓ RVHI, ANF	He et al. [105]
**MCT** (60 mg/kg) subcutaneous injection (rats)	4% H_2_ in air continuous inhalation for 21 days	↓ mast cells ↓ TGF-ß	Kuropatkina et al. [95]
2 mg/kg **doxorubicin** Every 3 days for 30 days IP injection (rats)	H_2_ saturated saline IP injection 10 mL/kg HRS daily for 30 days	↓ LVD ↑ EF, FS ↓ BNP ↓ ROS, MDA ↓ TNF-α, IL-6, IL-1ß ↓ Bax/Bcl-2 ↓ caspase 8, 3	Gao et al. [87]
2 mg/kg **doxorubicin** Every 4 days for 30 days IP injection (rats) doxorubicin (2 μM) (H9c2 cells)	2% H_2_ in air inhalation 6 h daily for 30 days H_2_ saturated medium	↓ HW/BW ↓ LVD ↑ LVEF, FS ↓ LDH, CK-MB, cTnI, BNP ↑ LC3, Beclin1, Atg7 ↓ Bax, cleaved caspase-3, 9 ↑ Bcl-2 ↑ p-AMPK/AMPK ↓ p-mTOR/mTOR	Ma et al. [103]
1% **adrenaline** (0.3 mg/kg) 3 times every 48 h IP injection (rats)	2% H_2_ in air inhalation 40 min a day for 5 days	↑ EPM, ATP, 2,3-DPG, CAT activity ↓ erythrocyte aggregation, MDA, RBC, Hg, MCV	Deryugina et al. [83]

## 5. Future Directions

Despite a growing number of studies confirming beneficial effects of H_2_ administration in different disease settings, its potential acceptance in clinics is still under development. The outcomes of several clinical studies have suggested that H_2_ can improve the consequences associated with cardiovascular diseases. These are mainly oriented on post-cardiac arrest syndrome [106,107] and myocardial infarction [108]. Nevertheless, clinical investigations in this area are still limited. Future large-scale and long-term trials are necessary not only to determine the clinical feasibility of using H_2_ as a therapy but also to verify its efficacy and safety. Different administration methods of H_2_ represent an advantage for its use in prevention or disease treatment. Oral consumption of H_2_-rich water is suitable for gastrointestinal disorders, whereas inhalation of H_2_ or injection of H_2_-rich saline is appropriate for systemic diseases. Inhalation of H_2_ is clinically more feasible compared with other methods of H_2_ delivery. Research in the area of H_2_ administration options is still evolving. For example, different nanomaterials and devices for H_2_ supply have been developed that aim to increase the concentration of H_2_ at the site of action and provide controlled H_2_ release and combined treatment [109]. Development of new delivery systems might be necessary to support the clinical potential of H_2_ therapy. The use of H_2_ in clinics presents several challenges. Manipulation with H_2_ during preparation, storage, transfer, and distribution of inhaled H_2_ requires attention to prevent potential explosions. Oral administration of H_2_ lacks targeted delivery to specific sites of action. Future research needs to explore the timing, dose–response relationship, and how H_2_ can be combined with other therapies. Finally, searching for the exact intracellular mechanisms of H_2_ action is needed as well to enable its transfer into clinical practice. With regard to HF, future research could be oriented around the investigation of H_2_ mechanisms to reduce ventricular dilation, decrease metabolic stress, or reverse adverse cardiac remodeling. Available in vivo studies of H_2_ effects in different HF models are performed on male animals only. Therefore, investigation of sex differences in the response to H_2_ treatment is also warranted.

## 6. Conclusions

Cardiovascular diseases are the leading causes of death worldwide and therefore warrant new therapies. Given the complexity of underlying pathological mechanisms, effective HF therapy requires a comprehensive approach. H_2_ is a small ubiquitous gaseous molecule that has been shown to exert diverse biological effects. Experimental and clinical studies demonstrate that the utilization of H_2_ can be beneficial in the prevention and treatment of various diseases, including HF. According to our knowledge, this is the first review summarizing experimental evidence for H_2_ effects in different HF models. The available literature indicates that H_2_ may be effective in mitigating different HF pathologies via regulating cardiac oxidative stress and inflammation, cardiomyocyte death and mitochondrial function/cell metabolism, as well as cardiac remodeling, including hypertrophy and fibrosis. Although the number of studies dealing with the effects of H_2_ in different HF models is continuously increasing, this area of research is still in its infancy. Further studies are needed to reveal exact intracellular mechanisms of H_2_ action, as well as its efficacy and safety for potential use as a novel treatment in HF.

## Data Availability

No new data were created or analyzed in this study. Data sharing is not applicable to this article.
